# The three dimensions of somatic evolution: Integrating the role of genetic damage, life‐history traits, and aging in carcinogenesis

**DOI:** 10.1111/eva.12947

**Published:** 2020-03-20

**Authors:** Andrii I. Rozhok, James DeGregori

**Affiliations:** ^1^ Department of Biochemistry and Molecular Genetics University of Colorado Anschutz Medical Campus Aurora Colorado; ^2^ Integrated Department of Immunology University of Colorado Anschutz Medical Campus Aurora Colorado; ^3^ Department of Pediatrics University of Colorado Anschutz Medical Campus Aurora Colorado; ^4^ Department of Medicine/Section of Hematology University of Colorado Anschutz Medical Campus Aurora Colorado

**Keywords:** carcinogenesis, life‐history evolution, longevity, somatic evolution

## Abstract

Tumors result from genetic and epigenetic alterations that change cellular survival and differentiation probabilities, promoting clonal dominance. Subsequent genetic and selection processes in tumors allow cells to lose their tissue fidelity and migrate to other parts of the body, turning tumors into cancer. However, the relationship between genetic damage and cancer is not linear, showing remarkable and sometimes seemingly counterintuitive patterns for different tissues and across animal taxa. In the present paper, we attempt to integrate our understanding of somatic evolution and cancer as a product of three major orthogonal processes: occurrence of somatic mutations, evolution of species‐specific life‐history traits, and physiological aging. Patterns of cancer risk have been shaped by selective pressures experienced by animal populations over millions of years, influencing and influenced by selection acting on traits ranging from mutation rate to reproductive strategies to longevity. We discuss how evolution of species shapes their cancer profiles alongside and in connection with other evolving life‐history traits and how this process explains the patterns of cancer incidence we observe in humans and other animals.

## 1 INTRODUCTION

Previous writings from our group, for example (Rozhok & DeGregori, 2015, 2016, 2019a) many other investigators (as referenced below), have provided deep insight into the processes controlling somatic evolution, with varying degrees of emphasis on mutational processes, selection, and the influences of the microenvironment. Still, we lack a clear understanding of the striking convergence of the age‐dependent patterns of cancer across tissues within a species and between species. Here, we will review the ideas and evidence that we have leveraged to propose an integrative model to explain the common age‐dependent pattern of cancers. This model proposes that cancer rates are dictated by three orthogonal evolutionary processes controlling somatic mutation occurrence, species‐specific life‐history traits, and rates of physiological aging. We propose that this model can reconcile what can appear as divergent observations in the field.

## 2 MUTATIONS, MULTIGENIC INHERITANCE, AND SELECTION

### 2.1 Carcinogenesis is defined by successions of mutant clones

**FIGURE 1:**
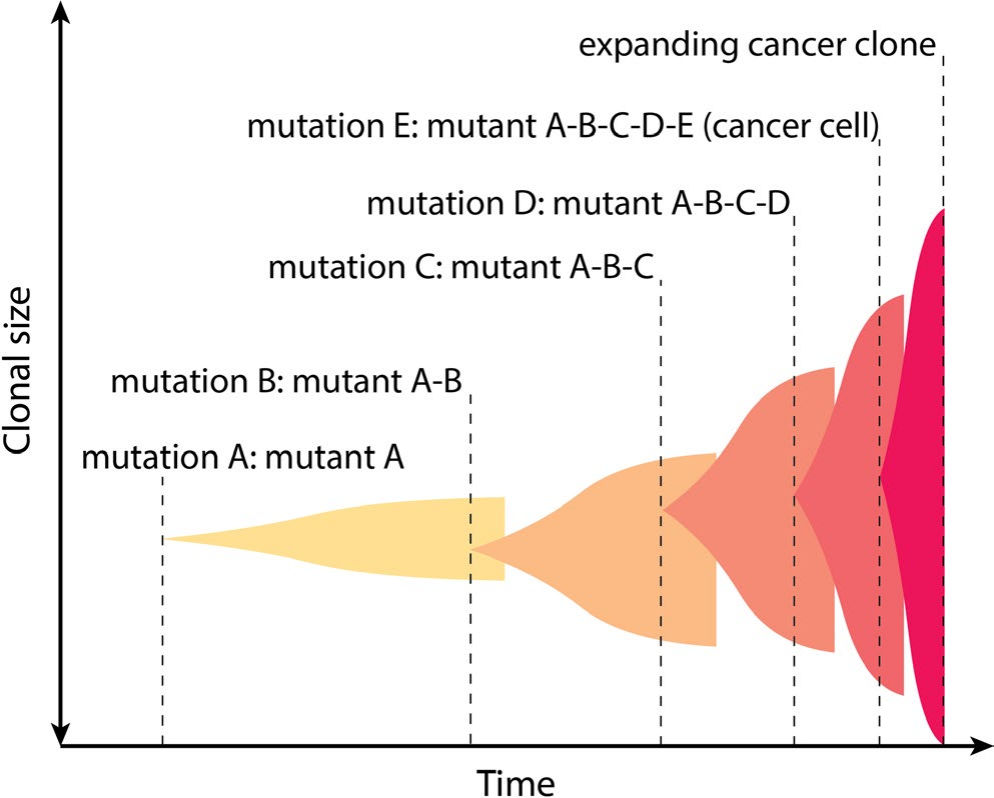
The multistage model of carcinogenesis. Shaded areas represent cell clones progressing toward cancer. The vertical width of every shaded area represents clonal size as shown by the *y*‐axis label. The *x*‐axis represents the timing of carcinogenesis. Mutations A, B, C, D, and E occur successively so that each next mutation occurs (for simplicity) in one cell of the previously expanded clone. Two critical changes develop over time: (a) Each successive mutation further elevates the relative fitness of the affected cell; therefore, the magnitude of clonal expansions increases at each successive stage of the process; (b) because greater clonal size represent a larger target for next mutations (increased the probability that the next mutation occurs in any cell of the clone), the time to the next mutation decreases, speeding up the process. A combination of these two processes will eventually generate an exponential curve of cancer incidence with age in a population

Cancer development depends on the accrual of genetic and epigenetic alterations (hereafter referred to in aggregate as mutations) that contribute to malignant phenotypes (Armitage & Doll, 1954; Nordling, 1953). Driven by somatic selection, oncogenically initiated cells expand into precancerous clones (Nowell, 1976). For an initially normally functioning cellular clone to expand and occupy a greater than expected fraction of a stem or progenitor cell pool, it needs to either increase the rate of cell division and/or decrease rates of differentiation or death. Further evolution of a rogue clone into a cancer requires the acquisition of other traits, including altered metabolism, promotion of angiogenesis, invasiveness, immune evasion, and metastatic spread (Hanahan & Weinberg, 2000, 2011). At advanced stages, cancer cells often contain a number of oncogenic mutations and sometimes exhibit mutator phenotypes that can provide more fuel for somatic evolution. The process of oncogenesis appears to be multistage in most cases, whereby multiple oncogenic events accumulate in a cellular clone sequentially and stepwise transform a cell into a progressively more cancerous phenotype. Usually, oncogenic events confer some fitness advantage to cells relative to other cells (initially normal, but later other tumor cells), leading to a series of clonal expansions. Each expansion increases the number of cells bearing a particular genetic context and therefore increasing the chances of further oncogenic transformations (Figure 1).

### 2.2 Mutation accumulation with age

Evidence for how somatic mutations accumulate in the body during life span varies. Comprehensive and well‐powered studies of DNA methylation changes with human aging reveal rapid accumulation of changes early in life before maturity when cell division rates are highest, with a subsequent reduced rate of change after maturity, such that roughly half of all such epimutations occur by maturity (Horvath, 2013). Similar results were obtained using a reporter gene to read out mutation accumulation in mice (Giese et al., 2002). However, more recent studies have shown a different pattern, at least for human hematopoietic stem cells (HSC), still with a more rapid accumulation of DNA mutations before birth, but with a clear linear pattern of accumulation from birth through old age (Osorio et al., 2018). Similar demonstrations of roughly linear mutation accumulation, albeit less well powered, were evident for other human tissues (liver, colon, and small intestine) (Blokzijl et al., 2016; Lee‐Six et al., 2018). It is interesting that DNA mutation accumulation is not more pronounced during prenatal ontogeny, given that more cell divisions should be required to generate the soma than to maintain it. Notably, Osorio et al. calculate that the per cell division mutation rate is substantially lower during HSC prenatal ontogeny compared to postnatal (Osorio et al., 2018), suggesting that natural selection could have developed mechanisms to limit mutation accumulation during ontogeny (while epimutations may more closely mirror patterns of cell divisions; Horvath, 2013). Another interesting observation from these recent studies of somatic mutation accumulation in stem cells is how lifetime mutation accumulation per stem cell is relatively similar across tissues—the rate of mutation accumulation per genome appears to be about 40 per year for liver, colon, and small intestinal stem cells, with roughly 3,000 mutations accumulating over 75 years (Blokzijl et al., 2016), despite the extremely different patterns of cellular turnover in these tissues. Similar mutation accumulation rates were evident for the colon in a different study (Lee‐Six et al., 2018) and for the endometrium (prepublication stage; BioRxiv, biorxiv.org; doi https://doi.org/10.1101/505685). These results raise the intriguing possibility that natural selection could have acted to limit mutation accumulation to some ceiling for different tissues, despite large differences in the mechanisms for tissue maintenance.

Whether front‐loaded or linear, cancer incidence does not follow either of these patterns of mutation accumulation, but reveals an exponential shape with most cancers delayed until later portions of the life span (Rozhok & DeGregori, 2019a). Two principal explanations currently exist to explain such a discrepancy. The first is that most cancers require multiple oncogenic mutations and, given the nature of carcinogenesis shown in Figure 1, the time when a cell in the body collects all of them is exponentially delayed (Armitage, 1985). This explanation appears logically valid. However, complex patterns of cancer incidence across animal taxa and tissues have raised doubts that cancer incidence can be explained by such a simple mathematical relationship. Therefore, an alternative explanation revolves around the idea that evolution has produced mechanisms that suppress carcinogenesis until later ages, and genetic damage accumulated early in life has little impact on organismal fitness through reproductive ages (DeGregori, 2011; Rozhok & DeGregori, 2015, 2019a).

### 2.3 Multiple mutations can lead to a cancerous phenotype

Working in concert with mutation occurrence, clonal expansion is absolutely critical for carcinogenesis, as this process multiplies the probability that several very rare events can occur in a single cellular clone (Nowell, 1976). Mutation rates are low, so collecting 3–6 rare mutations in one cell is extremely unlikely, unless the mutation‐bearing cellular clone is considerably expanded after each oncogenic event, markedly increasing the target size for the next mutation. Such enrichment is mostly achieved by selection acting on oncogenically initiated clones, favoring the spread of cells that possess better fitness parameters, the latter ultimately revolving around rates of cell division, differentiation, cell death, and senescence. Noteworthy, random drift can also lead to such an expansion without the action of selection (Vermeulen et al. 2013). Since the machineries underlying these processes are complex and thus encoded by many genes, there exist multiple ways to genetically transform a cell and endow it with a competitive advantage. For this reason, most cancers have a diversity of subtypes defined in part by different patterns of oncogenic mutations (Tomczak, Czerwińska, & Wiznerowicz, 2015). Genetic subtypes of cancers can be easily classified by sequencing. However, in order to confer a cell fitness advantage, mutations ultimately need to alter one or more of the fitness‐defining phenotypic traits, being the rates of cell division, differentiation, and death/senescence. While the genetic route to such phenotypes can be variable, there are genes and pathways that are recurrently mutated across cancers (e.g., the TP53 gene is mutated in more than half of all cancers; Hollstein et al., 1991).

### 2.4 Selection acts on phenotypes, not genotypes

At this stage, we should mention that natural selection does not act on genetic contexts directly. Selection reveals itself in the form of differential reproductive success of individuals in a population, whereby phenotypic traits that better match environmental demand are passed on over generations. The phenomenon of fitness is pivotal in determining selection, and thus, selection can only act on phenotype, since fitness is a phenomenon that arises at the interface of phenotype (not genotype) and the selective environment. In monogenic inheritance, whereby a trait is encoded by a single locus, there is a more rigid linkage between each particular gene and the phenotype the gene produces. However, the relationship becomes more complicated when a trait is encoded through multiple genes. A substantial portion of traits in animals and animal cells are encoded by multiple genes with varying contribution to the net phenotype (Boyle, Li, & Pritchard, 2017). In this case, only phenotype remains under direct action of selection, while particular genes experience the effect of selection based on their relative contribution to the selected phenotype. Low contribution to phenotype should lead an allele to effectively drift in a population. Evidence of such drift can be seen, for example, in analyses of sequences whereby the Kimura process of neutral evolution seems to have a major presence in many genes (Kimura, 1968). While the extent of Darwinian selection in the evolution of phenotypic traits is not currently fully understood, plentiful evidence of selection‐driven evolution at this level has accumulated (Ellegren, 2008). Many cancer mutations have also been shown to experience drift in tumors, selection being evident only in early initiating mutations ( Sun, Hu, & Curtis, 2018). However, unlike with mutations, selection toward more aggressive cancer phenotypes during tumorigenesis is most often clearly present in developing tumors.

### 2.5 Multigenic inheritance creates interindividual differences in mutation rates and cancer susceptibility

Mutation rate, just as many other traits related to cancer susceptibility, is a highly multigenic trait. As one indication, genetic screens for mutations affecting mutation rate in C. elegans revealed 61 genes whose disruption increased mutation rate, and almost all increased both germ line and somatic mutation rates (Pothof, 2003). As we have already argued and shown by stochastic modeling, multigenic inheritance ensures that the trait will not be uniform in a population, but will be variant and distributed (normally in a typical case), and the amount of variance relative to the mean is inversely proportional to the number of genes that encode a trait (Rozhok & DeGregori, 2019b). Following this logic and some evidence, mutation rates should vary in populations. In fact, mutation rates have undergone recent evolution in humans, leading to intrapopulation variation (Conrad et al., 2011; Harris, 2015; Harris & Pritchard, 2017). Therefore, interindividual variance in somatic mutation rate may impact cancer susceptibility. However, other physiological mechanisms exist that highly influence the effect of mutations on cells and cancer risk. These include traits such as autophagy (and other somatic maintenance mechanisms), the immune system (providing immune surveillance for potentially malignant cells and also influencing inflammatory responses), cell death/senescence (eliminating damaged or oncogenically mutated cells, but also impacting immune responses, tissue decline, and tumor development), and other tumor suppression mechanisms (Hanahan & Weinberg, 2000, 2011). Each of these traits is controlled by the products of many genes. Therefore, interindividual differences in cancer risk, being highly multigenic, will be expected to be normally distributed in populations, with a minor fraction of outliers. As such, we would expect that a small proportion of the population would be particularly susceptible to particular cancers, which is observed in humans (Foulkes, 2008; Hodgson, 2008). A marked exception from such patterns will be inherited cancer predisposition disorders that exhibit simple Mendelian inheritance (often with high penetrance; Foulkes, 2008), as these individuals often inherit at least one disrupted allele of a tumor suppressor gene, greatly increasing the chances that the additional necessary oncogenic events will occur in a single cell clone (often including the loss of the unaffected allele of the germ line‐mutated tumor suppressor gene).

The occurrence/accumulation of genetic damage and cell clonal expansions in tissues driven by mutations are therefore two fundamental underlying processes and the minimum condition for carcinogenesis to initiate. However, because selection does not act directly on genetic variants, but does so indirectly through acting on phenotype, and more so in multigenic traits, the relationship between particular mutations and cancer risk is not linear. We will further argue that cancer risk profiles typical of particular animal species are shaped by at least two more major evolutionary phenomena—life history‐dependent and species‐specific evolution of genes on the one hand and alterations in selective environment in tissues with age on the other.

## 3 LIFE HISTORY‐DEPENDENT AND SPECIES‐SPECIFIC EVOLUTION OF GENES AND CANCER PROFILES

### 3.1 Life histories add more to the story of carcinogenesis

The scenario of a multistage process of somatic evolution whereby each mutation drives clonal expansion and thus elevates the chances of the occurrence of each next mutation in any of the cells of the expanded clone is logically sufficient to explain why cancer incidence is exponentially delayed relative to the dynamics of mutation accumulation mentioned above. However, closer examination of patterns of cancer incidence across different bodily tissues and different animal species indicates that such a relationship is not the only mechanism determining age‐dependent carcinogenesis in real nature and most likely not the primary one (Rozhok & DeGregori, 2019a).

Perhaps the best known and most frequently discussed paradox in cancer incidence across species is Peto's paradox (Nunney, 1999a; Nunney & Muir, 2015; Peto, Roe, Lee, Levy, & Clack, 1975), whereby larger animals do not show higher frequencies of cancer. Given the greater number of cell divisions required to generate and maintain a larger body, often for longer life spans, large animals should have higher chances for the occurrence of oncogenic mutations throughout their life span. However, we do not observe the final result of carcinogenesis, cancer, more frequently in many studied large animals, such as whales (Griner, 1983). It is currently unclear, however, how many large animals demonstrate improved cancer resistance. According to some surveys, cancer is generally below 5% lifetime risk for most wild animals (Hochberg & Noble, 2017). However, much higher incidence of tumors has been reported for many various vertebrate species in the wild whenever animals survive into older ages (Madsen et al., 2017). More research is still needed to clarify what is the rate of such tumors developing into cancer with the ensuing cancer‐related mortality, the extent to which modern (often human‐instituted) changes in environments have altered cancer susceptibility, and what is the age distribution of such cancers. Combined, however, this evidence indicates that some mechanisms beyond the occurrence of oncogenic mutations affect cancer development.

Leonard Nunney was among the first to argue that an evolutionary solution to Peto's paradox could be in the independent evolution of cancer resistance mechanisms in different animals based on the detrimental effect of cancer in a specific group (Nunney, 1999b). Such an effect would certainly confer a life history‐dependent component in such mechanisms. The latter is also evident from the scaling of cancer incidence to species‐specific life spans (Rozhok & DeGregori, 2016), so that exponential rise in cancer incidence concurs with the latter portion of the life span, regardless of whether it is the 2‐ to 3‐year life span of a mouse or the 70‐ to 100‐year life span of a human. Mouse stem cells appear to divide faster than human (e.g., Sykes & Scadden, 2013 for HSC), but there are much fewer of them, and the animal life span is much shorter. There are many other species‐specific differences, from cell‐intrinsic elimination of cells with oncogenic activation to the maintenance of tissue integrity to immune function, which could contribute to the relative risks of cancer. It could have been a coincidence that the differences between the mouse and human stem cell parameters and myriad other tumor suppression systems are such that they roughly equalized cancer risk for both species as a function of age relative to life span. However, such scaling of cancer incidence to life span is observed in many animal species (Albuquerque, Drummond do Val, Doherty, & Magalhães, 2018), recognizing that data for other species are more limited. This leads to the question of what evolutionary forces could have driven species‐specific tumor‐suppressive systems to tune various parameters so that each species has a similar profile of age‐dependent cancer? Because the pattern is ubiquitous among animals, the chance of coincidence is negligibly low, and a likely explanation is that the evolution of life‐history traits has a general mechanism that postpones cancer to postreproductive periods of life spans in a species‐specific manner. For wild animals, by “postreproductive period,” we refer to ages at which most animals do not survive for any reason, as individual fitness rooted in the efficiency of reproduction is not visible to selection during such ages which are thus postreproductive de facto.

**FIGURE 2:**
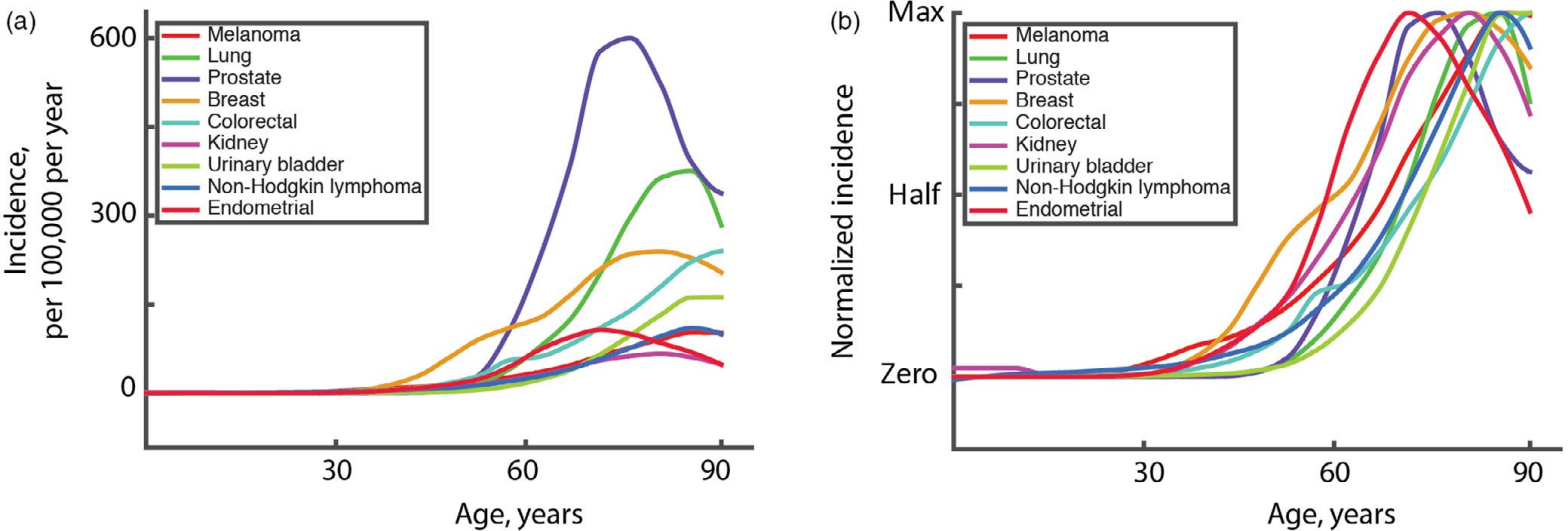
The incidence of the top nine most common cancers in the United States in 2012–2016 according to the National Cancer Institute data (www.seer.cancer.gov). (A) Absolute incidence per 100,000 people per year depending on age (top nine cancers, excluding nonmelanoma skin cancers, are plotted). (B) Normalized incidence obtained by first subtracting the minimum number of each number in the dataset (positions lowest incidence to zero on the Y‐scale) and then dividing the resulting values at each age for a given cancer by the maximum incidence of that cancer (removes the vertical scale)

Natural selection has acted to promote survival to ages sufficient to maximize reproductive success, which has entailed preventing early deaths from cancer. For this mechanism to function, cancers would need to be similarly delayed across tissues, as less effective tumor suppression for one particular tissue would render more effective suppression for other tissues a waste. Along these lines, somatic selection processes in different tissues of the body provide another line of evidence for why clonal succession in the multistage process of carcinogenesis deviates from the timing of oncogenic mutation occurrence. Cancers in different human tissues demonstrate a remarkable synchrony in their age dependence whereby the vast majority of cancers demonstrate a notable rise in incidence beginning at roughly the same age (data of National Cancer Institute; www.seer.cancer.gov), as shown in Figure 2. Yet, those cancers require vastly different numbers of oncogenic mutations, ranging from 1 to at least 10 (Rozhok & DeGregori, 2019a). Even late‐life clonality in some tissues, such as hematopoietic, apparently driven by a single mutation, follows faithfully the exponential cancer curve (Jaiswal et al., 2014; Martincorena, 2019). Moreover, there is more complexity added when we consider the vastly different organization of stem cell compartments in different tissues and very different selection processes in them. For example, selection is known to be dominant in governing the fate of oncogenic mutations in the blood system (Biasco et al., 2016; Chung & Park, 2017). Yet in epithelial systems, such as the gut epithelia, the substantial presence of drift has been found. Even mutations in the TP53 gene, Kras activation and loss of APC that are powerful drivers of clonal expansion in other tissues and almost omnipresent in a large portion of cancers had significantly reduced ability to promote selection and clonal expansion in gut epithelia. A measurable presence of selection for TP53 loss‐of‐function mutants in the gut epithelia only appears under certain conditions, such as increased inflammation known to promote carcinogenesis (Vermeulen et al. 2013). Given thus the diversity of genetic damage required to promote particular cancers, the vastly different organization of tissues stem cell compartments, and the differences in selection/drift thresholds in various tissues, the synchronized timing in age‐dependent incidence of various types of cancers is unexpected.

There is a significant lack of power in cross‐species comparison currently due to lack of studies and a rather limited list of species for which cancer statistics are known. However, interesting patterns can be observed among rodents. The house mouse (*Mus musculus*) is a small and short‐lived species weighing 20–30 g with the physiological life span of up to 2–3 years. Most mice do not survive in nature past 1 year. Judging by size and longevity, mice should be at low risk of cancer. However, in captivity, whereby mice can survive into old ages, they reveal rather higher susceptibility to cancer, with 50%–90% of old mice dying of cancer (Ikeno et al., 2009; Lipman, Galecki, Burke, & Miller, 2004; Szymanska et al., 2014; Ward, 1983). One caveat to this statistic, though, is that it is unclear how much the highly inbreed nature of laboratory mice contributes to cancer susceptibility. The naked mole rats (*Heterocephalus glaber*) are small rodents of similar body weight (30–35 g); however, these live into ages past 30 years (Azpurua & Seluanov, 2012). Alongside their longevity, naked mole rats are known to be highly resistant to cancer (Buffenstein, 2008; Liang, Mele, Wu, Buffenstein, & Hornsby, 2010), and cancers are rarely observed in these rodents (at least until the ages past 30 years that they have been observed in captivity). Similarly, up to such ages no signs of physiological aging were observed (Buffenstein, 2008). A number of mechanisms for cancer resistance have been proposed for this species, including improved control over cell division (Seluanov et al., 2008) and the presence of an altered form of hyaluronic acid (Tian et al., 2013). However, other related species have been examined, such as *Spalax golani* and *S. judaei*, with comparably high resistance to cancer, with the proposed mechanisms of cancer resistance that differ from *H. glaber* (Gorbunova et al., 2012). The extent to which these proposed mechanisms contribute to cancer resistance is still not established for these three mole species. However, incidence patterns in these rodents do exhibit a commonality with other mammals—the onset of high cancer incidence in mice and naked mole rats is not observed through the ages of high somatic fitness (“youthfulness”). Capybara (*Hydrochoerus hydrochaeris*) represents another studied example from the order Rodentia. Capybara are the largest living rodents, attaining weights above 70 kilograms and with a physiological life span of 8–10 years (but not typically surviving past 4 years in the wild). Emerging research (currently at the prepublication stage) has found mechanisms of improved cancer resistance in capybara through enhanced T‐cell‐mediated tumor suppression (BioRxiv; biorxiv.org; doi: https://doi.org/10.1101/424606). Finally, recent studies have shown how larger and longer‐lived rodents like the beaver exhibit SIRT6 alleles that improve double‐stranded DNA break repair (Tian et al., 2019). Notably, the efficiency of double‐stranded break repair correlated with longevity across rodent species, not body size. Although data are still scarce, the emerging patterns are in agreement with the assumption that extended longevity promotes the evolution of improved tumor suppression machinery.

### 3.2 Life history‐dependent selection explains the evolution of cancer‐related genes

The evidence discussed above raises the question of what mechanisms could explain such a universal pattern of age‐dependent cancer incidence across bodily tissues and animal taxa? As we have argued previously (Rozhok & DeGregori, 2019a), there is a straightforward evolutionary mechanism that should underly this phenomenon. This mechanism lies in the fact that similar genes undergo vastly different evolutionary paths in different species, including the cancer‐related genetic machinery. The overarching process in animal evolution is the environment‐driven adaptation of species to various ecological niches, which imposes selection for very different life‐history traits, such as body size, longevity, and reproductive parameters. Longevity and the curve of physiological aging, in particular, evolves based on the probability of physical survival and reproduction into certain ages due to factors such as predation, diseases, and other ecological factors (Hamilton, 1966). If this probability changes, species react with corresponding changes in the curve of physiological aging (Austad, 1993; Bryant & Reznick, 2004; Williams & Day, 2003). Physiological aging can be observed in modern humans and captive animals as the exponentially increasing probability of disease and death late in life, often at ages that are rarely reached “in the wild.” This process, in turn, imposes changes in selection acting on genes, particularly on genes that can cause premature death, such as cancer‐related genes. This mechanism ultimately operates through reproductive success, such that a mutation that causes death before the reproductive period is over (usually through all or most of the period of physical survival in the wild) reduces the affected individual's overall reproductive success relative to others in the population. Reduced reproductive success triggers selection that over time eliminates such a genotype from the population.

It is thus logical to conclude that the genes in multiple pathways critical for oncogenesis and tumor suppression have undergone vastly different evolutionary paths in animals with different longevity. An oncogenically altered gene that kills a mouse at the age of 3 years will have a much lower, if any, impact on the overall reproductive success of that mouse compared to one that kills a dog, human, or a whale at the same age. In longer‐lived species, selection will act much more strongly to buffer the effect of mutations in that gene to ensure survival through reproductive ages. Similarly, larger bodies will increase the risk of oncogenic mutations per body per lifetime and thus will add selection pressures to lower the ensuing risk of cancer‐related death. As a result, in the absence of species/group specific mechanisms, the overall multigenic tumor‐suppressive machinery should vastly differ in species with different longevity and body size. Since mutations are random, with comparable mutation rates various animals should be under similar age‐dependent cancer risk normalized to body size and longevity (larger and longer‐lived animals should have proportionally higher risk of cancer). However, due to the vastly different species‐specific evolution of genes controlling cancer risk, the buffering capacity of tumor suppression machinery in a given species is simply as high as is needed to survive through the reproductive period of this particular species. An interesting correlation that agrees with this logic has been observed for humans, revealing that the frequency of alleles that predispose to higher cancer risk in human populations is inversely proportional to how much they elevate cancer risk (Foulkes, 2008). This pattern is consistent with selection pressures acting on dangerous alleles being modulated by the lifetime risk conferred by those alleles. It would be very informative to compare the population frequencies of such alleles in other animals; however, such studies are still lacking to the best of our knowledge. Notably, improvements in the efficiency of tumor suppressor machinery could also be hampered by the cost. For example, autophagy is believed to be energetically expensive (Rabinowitz & White, 2010). In the natural conditions of limited food supply, therefore, improved autophagy might negatively impact fitness even when cancer risk is reduced.

Tumor suppressor machinery of a particular species is hardwired by evolution of that species in such a way that the likelihood that any given mutation will have an effect sufficient to kill an individual of that species prematurely is very low. The same mutation in a different species can have a different effect. Experiments show, for example, that different numbers of oncogenic mutations are needed to malignantly transform the same cell types of different species (Hamad, 2002; Rangarajan, Hong, Gifford, & Weinberg, 2004), as well as different cell types of the same species (Oldham, Clark, Gangarosa, Coffey, & Der, 1996). This evidence elucidates the vastly discrepant evolution of genetic machineries in different species. However, while a particular gene will typically be the same in different tissues of the same individual, it is the proteomic network, expression profiles, and tissue‐specific mRNA splicing that regulate the function of each protein in particular tissues and that differ among tissues. These differences underlie the variability in the effect of particular mutations on cancer risk for specific tissues. So, it is tempting to assume that evolutionary trade‐offs help explain scenarios whereby a particular allele that confers higher cancer risk in some tissues is retained by selection because this allele is important for the function of other tissues and thus improves the overall fitness of the organism. If that has been the case in animal evolution, it could explain one of the mechanisms that prevented a more effective elimination of cancer risk by natural selection.

Therefore, the dynamics of the multistage process of successive oncogenic mutations and the ensuing clonal expansions leading eventually to cancer is controlled by substantially different genetic machineries in different species, resulting in different delays between the occurrence of oncogenic mutations and cancer. However, due to data scarcity, it remains unclear what portion of the total tumor suppressor and tissue maintenance machinery is common across taxa and what portion is group specific. While common mechanisms clearly exist, such as autophagy and the immune system, it is possible that when a group/species‐specific mechanism evolves in a particular group that significantly lowers cancer risk, selection on other mechanisms may relax, resulting in different degrees of the efficiency of common mechanisms in different groups. While common mechanisms could be the primary tumor suppressor machinery in some or most species, it appears that some species employ mechanisms that do not exist at all in other taxa. Studies on rodents discussed above present some early evidence of this possibility.

### 3.3 Genetic machinery underlying cancer suppression is inherently imperfect

An important theoretical question that stems from the described processes is why some cancers still occur early in life. We have already mentioned that evolutionary trade‐offs among organismal tissues could point to one reason. Another straightforward answer would be that evolution has not had enough time to eliminate such a possibility completely, particularly when such cancer risk is very low (and thus selection to eliminate such risk will be weak). However, an additional mechanism should be operative that should preclude the elimination of risk‐conferring alleles and their “visibility” to selection. And this mechanism resides in multigenic inheritance. As we have mentioned above, selection can only act on genes directly when the selected trait is encoded by one gene. However, many traits, including mutation rates and susceptibility to cancer, are multigenic. The evolution of genes encoding multigenic traits, therefore, depends on the contribution of a particular gene to the trait. Genetic alleles with higher impact will be under stronger selective pressures, while those with minor contribution will likely evolve by drift. More complexity is added by considering that many genes contribute to multiple traits. For example, the activity of the p53 protein is strongly linked with cancer susceptibility (Hollstein et al., 1991; Stracquadanio et al., 2016). However, p53 also controls the tissue regenerative function of stem cells by directing stem cells into apoptosis when a certain threshold level of genetic damage occurs in the cell. When a cell dies after a low amount of DNA damage, cancer risk might be lowered, even if marginally. However, premature apoptotic signaling could lead to depletion of the stem cell compartment and likely earlier aging. Premature aging lowers reproductive success and fitness, just like higher cancer susceptibility does. Therefore, the activity and architecture of p53 are likely tuned by evolution so that it maximizes fitness in a particular species. Therefore, selection acting on p53 will be balanced in a way that some cancer risk remains present. An example of such a balance in selection acting on p53 could potentially come from recent studies that proposed roles for TP53 gene evolution in cancer resistance in elephants (Abegglen et al., 2015; Sulak et al., 2016). A total of 20 copies of the TP53 genes were found in elephants, which the authors hypothesize, could explain higher cancer resistance through the p53‐mediated pathways. Most of the 19 TP53‐derived retrogenes lack clear functional roles, while at least one of these genes was shown to function in a manner consistent with improved tumor suppression (Sulak et al., 2016). It is still unclear the extent to which the extra copies of TP53 in elephants contribute to cancer resistance (testing such a hypothesis, at least in elephants, is difficult). But the fact that most of the extra copies are likely functionally inactivated suggests either that selection has rather disfavored multiple TP53 copies in elephants, potentially due to the discussed above trade‐offs higher levels of p53 impose, or that these additional copies were insufficiently favored so as to be under purifying selection. More research is needed to clarify this potential mechanism of cancer resistance in elephants (e.g., would the truncated elephant allele confer cancer resistance in rodents?).

A population will usually harbor multiple alleles for many genes that combined determine susceptibility to mutations and cancer. Cancer susceptibility is usually of multigenic origin, since tumor suppression involves multiple pathways and genes. A hypothetical allele A of gene 1 may increase cancer risk if present in the same genome with a hypothetical allele B of gene 2 or allele C of gene 3. If allele 2B increases individual fitness via its effect on other traits and allele 3C is fitness‐neutral, then allele 1A will be under negative selection in individuals with genotype 1A2N3C (N for any other allele). However, in 1A2B3N individuals, selection acting on allele 1A will be balanced by the opposite direction of selection acting on allele 2B. Thus, there will be a distribution of fitness effects for 1A across individuals in the population, with the fitness effects in each individual determined by what other alleles are encoded in the same genome with 1A. Importantly, if allele 1A increases fitness in most genetic contexts, selection will likely retain the allele in the population regardless of its fitness‐reducing effect in a minor fraction of the population. Such context dependence in how selection acts on genes encoding multigenic traits and genes contributing to multiple traits will result in selection acting on one and the same allele differently in different individuals of a population, which will leave some risk‐conferring alleles in a population regardless of the risk they confer. In such a scenario, it is possible that selection is simply uncapable of perfecting tumor suppression machinery to the point of preventing all cancers that curtail reproductive success, as it cannot “see” all instances of a particular allele in a population.

Based on the concepts described above, we can conclude, therefore, that when other animal taxa and multiple cancer types are considered and compared from a broader evolutionary perspective, and considering how genes evolve across animal taxa, it appears to be the species and life history‐specific evolution of genes that determines the effect of any given mutation on the cellular machinery and therefore the magnitude of mutation‐driven clonal dynamics. As we have argued above, such effects can also be tissue specific. The architecture of tissues and cellular machineries evolving in such a complex way will determine the time‐dependent probabilities of the next mutations in a sequence and then ultimately the timing of cancer. In this way, age‐dependent incidence of cancer should be primarily determined by the overarching process of the evolution of species‐specific life‐history traits and ultimately the curve of physiological aging which mostly evolves based on ages of likely physical survival in the wild.

## 4 THE NATURE OF SOMATIC SELECTION: WHY PHYSIOLOGICAL AGING SHOULD PROMOTE SOMATIC EVOLUTION

### 4.1 Aging alters the fate of mutations in the soma

We described in the previous section how the hardwiring of the genetic machinery by evolution over long periods of times impacts cancer risk across individuals and species. This process modifies the effects of particular mutations on cells and tissues, and thus determines the probability over time for an individual of a given species to acquire specific mutations in the quantity sufficient to malignantly transform a cell. This probability should significantly impact the age dependence of the incidence of most cancers. However, the curve of physiological aging should itself impose dramatic shifts in somatic selection and the effects of mutations over lifetime. In order to understand why physiological aging should promote somatic evolution, we first need to briefly overview how environment affects selection.

**FIGURE 3:**
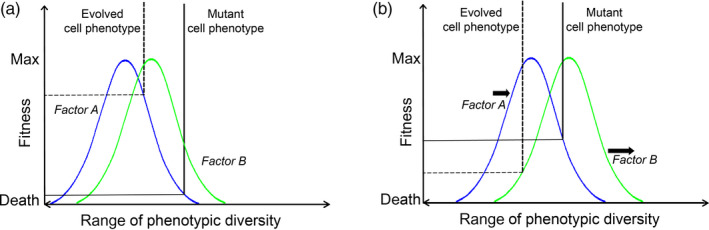
Determination of fitness in the Sprengel–Liebig system. (a) The fitness of a phenotype along the range of the phenotypic variation relative to a certain environmental factor is typically distributed normally following Shelford's law of tolerance (Rozhok & DeGregori, 2015), with a certain value of the expression of a phenotype being optimal (highest fitness) and fitness decreasing progressively in phenotypes that deviate more from the optimal expression (pessima). In multifactorial environments, the fitness of a phenotype will be limited by the factor to which the phenotype is most poorly adapted. Here, factor A (blue curve) is the fitness‐limiting curve for both the evolved and mutant phenotypes. The evolved phenotype has higher fitness. (b) Changes in environment shift the distributions of phenotypes relative to environmental optima and, respectively, phenotype‐altering mutations change the position of the resulting phenotype relative to environmental optima. Both processes can alter both the fitness of a phenotype and the factor that limits the phenotype's fitness. Here, an environmental shift in factors A and B has led to the mutant phenotype gaining fitness advantage of the evolved phenotype. The factor that limits the fitness of the evolved phenotype has also changed. This figure is adapted from (Rozhok & DeGregori, 2015)

A phenotype usually consists of multiple traits. Each phenotypic trait is usually distributed in a population, with the population mean being typically the most frequent expression of the trait. These distributions differ in the amount of variation, depending on how many genes encode the trait, the strength of purifying selection acting on the trait, and other factors. Typically, a normal distribution characterizes a well‐adapted population whereby the frequency of phenotypes (and their fitness) decreases toward larger deviation from the mean. Selection optimizes each trait in a way that ensures the highest possible net fitness of an individual. The individual contribution of each phenotypic trait affects the net organismal fitness. However, individual fitness is often determined by a phenomenon observed independently by Sprengel and Liebig and dictating that individual fitness will be limited by an environmental factor (such as a nutrient) that is most restricted, regardless of the abundance of other factors (summarized in Gorban, Pokidysheva, Smirnova, & Tyukina, 2011). Likewise, the net fitness of an individual will be limited by the trait that confers the poorest adaptation to the current environment. As we have argued previously (Rozhok & DeGregori, 2015), because both the phenotype and the environment are complex (multifactorial), a change in the environment often not only leads to selection optimizing the key traits of a phenotype, but often alters the traits that are under strongest selection. As shown in Figure 3, this occurs because the fitness‐limiting trait may change following changes in the environment. An illustration could be made that if the hair color of a snowshoe hare does not match environmental demand (the white color of snow in the winter and gray color in the summer), fur color could become the main trait under selection as it provides the most dramatic differences in relative survival. However, once the alleles providing white/gray fur are fixed in the population, relative survival could become more dependent on other traits, such as the number of progeny in litters or a better ability to utilize sources of nutrition in the current environment, depending on which trait provides the largest survival differential and leading to the spread of the underlying alleles. In nature, such selection changes are gradual rather than abrupt. However, systemic alterations in complex environments will often make it difficult to predict how exactly selection will change. The example in Figure 3 illustrates the reason.

Stem cell compartments of animal tissues are frequently the place where early precancer somatic evolutionary processes begin, with sometimes substantial clonal expansions preceding carcinogenesis. Stem cells are known to compete for the limited stem cell niche space in tissues (Abkowitz, Catlin, & Guttorp, 1996; Abkowitz, Golinelli, Harrison, & Guttorp, 2000; Vermeulen et al. 2013). Their relative clonal dynamics are governed by cell division, differentiation, senescence, and death. These behaviors are regulated by extracellular signaling coming from the stem cell niche environment and have been fine‐tuned by evolution for optimal tissue maintenance. This process required co‐adaptation, through evolution over millions of years, of the cellular machinery of stem cells and the signaling coming from the tissue microenvironment. By definition, such a co‐adaptation should have been species‐specific and possible only for the portion of the life span during which individuals can survive and reproduce in nature, since reproduction is the ultimate currency of selection. Degradation processes that occur in tissues during physiological aging at ages most animals do not survive in the wild lead to profound alterations in tissue microenvironments, such as increased inflammation, the accumulation of senescent cells, and changes in extracellular matrix (Phillip, Aifuwa, Walston, & Wirtz, 2015). Since behavioral parameters of stem cells have not been optimized for such environments, based on the theory shown in Figure 3, aging tissues will engender unpredictable changes on the relative fitness of specific cell genotypes, including those cells bearing oncogenic mutations. Here, we should note that the influence of aging on the cell somatic fitness of many specific somatic cell phenotypes is known. The term “unpredictable” here is rather used as a general theoretical concept when the entire diversity of somatic cell phenotypes is considered. The effect of aging on the cell somatic fitness has only been explored experimentally for a very small subset of the somatic cell diversity of the body. Just as in natural populations, such changes should lead to substantial reduction in the amount of stabilizing selection acting on stem cells (such as the stabilizing pressure of the aggregate tumor‐suppressive machinery) and promote directional selection and clonal selective sweeps that are necessary for the multistage process of carcinogenesis. Evidence of increased positive selection and selective sweeps in aged tissues comes, for example, from studies of clonality in the hematopoietic system, whereby aged bone marrow harbors substantially expanded hematopoietic stem cell clones driven by a single mutation (Bowman, Busque, & Levine, 2018).

Experimental evidence has accumulated over the last decade that corroborates the theory just discussed and shows that somatic selection acting on the same mutations is substantially altered by aging‐related processes (Henry et al., 2015; Marusyk & DeGregori, 2008; Vas, Wandhoff, Dörr, Niebel, & Geiger, 2012; Vermeulen et al. 2013). The immune system has been demonstrated to be another critical mechanism promoting purifying selection in tissues by directly culling oncogenically initiated cells and thus preventing their clonal proliferation and further stages of carcinogenesis (Swann & Smyth, 2007). An important role of autophagy has been shown as well (Green & Levine, 2014). Manipulation of major autophagic pathways in mice through such genes as BECN1 and ATG5 has shown that improved autophagy simultaneously extends longevity and improves cancer resistance (Fernández et al., 2018; Pyo et al., 2013), revealing thus another link between aging and somatic evolution. These tumor‐suppressive systems are also known to undergo significant alterations as a function of age. As a result of such alterations, the survival chances and fitness of many cancer cells should also increase into older ages.

We thus conclude that, in conjunction with the process of mutation incurred by cell division and exposures and the species‐specific evolution of tumor suppression machinery, physiological aging is another major process that adds complexity to the cancer equation, specifically the age‐dependent incidence of cancers.

## 5 CONCLUSION

A number of models and theoretical frameworks for explaining age‐dependent incidence of cancer and the process of carcinogenesis have been proposed to date, with the overarching theory postulating carcinogenesis as a series of genetic and epigenetic transformations in a cell leading to a malignant cell phenotype. Clonal proliferation driven by each of such changes expands the affected cellular context providing an increased target for subsequent oncogenic events in cells, until cancerous cells finally appear over time. Importantly, most genetic mutations do not have a defined fitness effect in natural populations and thus cannot promote a specific predicted mode and strength of selection, but their effect is rather substantially determined by the current selective environment and depends on particular genetic contexts in which these mutations occur. Likewise, it is logical to assume that while mutations are the ultimate fuel of carcinogenesis, it is the selective environment in tissues and the genetic machinery hardwired by long‐term evolution in particular species that determine the mode and strength of somatic evolution they drive. Evolutionary vision adds an entire dimension to our understanding of cancer. For example, the paradox of larger animals not demonstrating higher frequencies of cancers seems like a paradox only if looked at from a fixed time point, or current state, perspective. However, we can consider that both small and large animals have undergone a long period of evolving antitumor machinery in a species‐specific way with very different selective pressures acting on their tissue maintenance machineries and specific genes based on their species‐specific risk factors (such as a large body). From this perspective, it becomes clear that species‐specific cancer profiles are a function of maximized reproductive success, rather than body size. Much more extensive evaluation of cancer incidence and timing in wild animal populations will be required to test these predictions. Moreover, it becomes clear that the tumor suppression machinery is just good enough to allow an individual to survive through its species‐specific ages of reproduction, but not substantially beyond this period. Just like aging and the incidence of many other diseases, cancer incidence should primarily be explained by the relaxed germ line selection controlling fitness in advanced ages and the ensuing relaxed somatic purifying selection in tissues following tissue degradation with age. These predictions should spur additional studies to test how aged tissue environments influence selection for oncogenic phenotypes and the consequent impacts on cancer rates with age.

Modern evidence, therefore, allows us to argue that somatic evolution and its partial case, carcinogenesis, are shaped by three major orthogonal processes: accumulation of somatic mutations over lifetimes, species‐specific evolution of cellular genetic machinery, and physiological aging‐induced shifts in selective microenvironments in tissues. The three processes are interconnected through the evolution of life‐history traits and therefore should vastly differ across species, with the common denominator being species‐specific reproductive success and the evolution of aging.

## CONFLICT OF INTEREST

None declared.

## Data Availability

As this is a review, no data are described that require archiving.
